# Alpha-Band Oscillations Reflect Altered Multisensory Processing of the McGurk Illusion in Schizophrenia

**DOI:** 10.3389/fnhum.2016.00041

**Published:** 2016-02-12

**Authors:** Yadira Roa Romero, Julian Keil, Johanna Balz, Michael Niedeggen, Jürgen Gallinat, Daniel Senkowski

**Affiliations:** ^1^Department of Psychiatry and Psychotherapy, Charité–Universitätsmedizin Berlin – St. Hedwig HospitalBerlin, Germany; ^2^Department of Education and Psychology, Free University BerlinBerlin, Germany; ^3^Department for Psychiatry and Psychotherapy, University Medical Center Hamburg-EppendorfHamburg, Germany

**Keywords:** schizophrenia, neural oscillations, multisensory integration, audiovisual, speech

## Abstract

The formation of coherent multisensory percepts requires integration of stimuli across the multiple senses. Patients with schizophrenia (ScZ) often experience a loss of coherent perception and hence, they might also show dysfunctional multisensory processing. In this high-density electroencephalography study, we investigated the neural signatures of the McGurk illusion, as a phenomenon of speech-specific multisensory processing. In the McGurk illusion lip movements are paired with incongruent auditory syllables, which can induce a fused percept. In ScZ patients and healthy controls we compared neural oscillations and event-related potentials (ERPs) to congruent audiovisual speech stimuli and McGurk illusion trials, where a visual /ga/ and an auditory /pa/ was often perceived as /ka/. There were no significant group differences in illusion rates. The EEG data analysis revealed larger short latency ERPs to McGurk illusion compared with congruent trials in controls. The reversed effect pattern was found in ScZ patients, indicating an early audiovisual processing deficit. Moreover, we observed stronger suppression of medio-central alpha-band power (8–10 Hz, 550–700 ms) in response to McGurk illusion compared with control trials in the control group. Again, the reversed pattern was found in SCZ patients. Moreover, within groups, alpha-band suppression was negatively correlated with the McGurk illusion rate in ScZ patients, while the correlation tended to be positive in controls. The topography of alpha-band effects indicated an involvement of auditory and/or frontal structures. Our study suggests that short latency ERPs and long latency alpha-band oscillations reflect abnormal multisensory processing of the McGurk illusion in ScZ.

## Introduction

Numerous studies using auditory (Leavitt et al., 2007; Rosburg et al., 2008; Popov et al., 2011) or visual stimuli (Butler et al., 2007; Tan et al., 2013) have shown perceptual deficits in schizophrenia (ScZ). Recently, perceptual processing in ScZ has also been investigated in multisensory setups, (Ross et al., 2007; Williams et al., 2010; Stone et al., 2011; Stekelenburg et al., 2013), but findings were less consistent than in unisensory studies. Multisensory processing requires the coordinated integration of information across widespread cortical areas, which is presumably impaired in ScZ (Stephan et al., 2006; Uhlhaas and Singer, 2006). The coordination of information across brain areas likely involves neural synchronization, expressed in oscillatory activity (Fries, 2005; Senkowski et al., 2008). While previous research using unisensory stimuli has provided strong evidence for abnormal oscillatory activity in ScZ (Spencer et al., 2008; Uhlhaas and Singer, 2010; Grützner et al., 2013; Popov et al., 2014), currently only one patient study has examined oscillatory activity in multisensory processing (Stone et al., 2014). In a multisensory detection task, the authors observed altered gamma-band activity (i.e., 30–50 Hz) in ScZ patients.

An experimental paradigm that is well suited to examine multisensory processing is the McGurk illusion (McGurk and MacDonald, 1976). This illusion is found when lip movements pronouncing a syllable (e.g., /ga/) are paired with incongruent auditory syllables (e.g., /ba/). The pairing of specific incongruent visual and auditory syllables can induce a fused percept (e.g., a visual /ga/ and an auditory /ba/ is often perceived as /da/). Thus far, only few studies have examined the McGurk illusion in ScZ. Some studies reported a reduced McGurk illusion rate in ScZ patients compared with controls (Gelder et al., 2002; Pearl et al., 2009; White et al., 2014). However, a recent study found no group differences in illusion rates (Martin et al., 2013). Notably, previous studies using non-McGurk type audiovisual speech stimuli in ScZ also revealed inconsistent results (Surguladze et al., 2001; Szycik et al., 2009; Stekelenburg et al., 2013). For instance, Surguladze et al. (2001) used word stimuli to examine audiovisual speech perception and found no differences in susceptibility for the fusion perception between ScZ patients and healthy controls. In contrast, Stekelenburg et al. (2013) found ERP differences in the processing of congruent and incongruent audiovisual speech. Hence, further research is required to examine multisensory processing in ScZ.

In this high-density electroencephalography (EEG) study, we investigated the McGurk illusion in ScZ patients and matched control participants. Recently, we observed that neural oscillatory activity play a role in the McGurk illusion in healthy participants (Roa Romero et al., 2015). Hence, we hypothesized that oscillatory activity, reflecting the processing of the McGurk illusion is altered in ScZ patients. Here, we investigated effects across a broad frequency range of 4–40 Hz. Moreover, we examined possible interactions in event-related potentials (ERPs).

## Materials and Methods

### Participants

Twenty-one patients with the DSM-IV diagnosis ScZ were recruited from outpatient units of the Charité–Universitätsmedizin Berlin. In addition, 21 age, education, and handedness matched healthy control participants, who were screened for mental disorders with the German version of the Structured Clinical Interview for DSM-IV-R Non-Patient Edition (SCID), participated in the study. Due to a lack of McGurk illusion perception (i.e., illusion rate < 15%, ScZ patients = 5; matched controls = 5) and insufficient EEG data quality (ScZ patients = 2; matched controls = 2), data from seven ScZ patients and seven matched control participants were excluded. The illusion rates of excluded subjects did not significantly differ between groups (Mann–Whitney *U* test = 24, *p* = 0.95). All patients fulfilled the DSM-IV-TR and ICD 10 criteria for ScZ and no other axis I disorder. The psychiatric diagnosis was assessed by a senior psychiatrist at the recruiting institution. All participants had normal hearing, normal or corrected to normal vision, and no neurological disorders, alcohol or substance abuse. A random sample of 45% of all participants underwent a multi drug screening test. None of the tested participants had a positive test outcome. Severity of symptoms in ScZ patients was assessed with the Positive and Negative Syndrome Scale (PANSS; Kay et al., 1987). To test cognitive performance, the Brief Assessment of Cognition in Schizophrenia (BACS) was assessed (Keefe et al., 2004). **Table 1** provides an overview on demographic data, cognitive performance, and clinical scores. All participants gave written informed consent in accordance with the Declaration of Helsinki. The local ethics commission of the Charité–Universitätsmedizin Berlin approved the study.

**Table 1:** Overview of demographic data.

	Patients		Controls		Statistics	
			
	Mean	*SD*	Mean	*SD*	*t*-values	*p*-values
Age (years)	35.57	6.55	36.79	7.18	–0.468	0.664
Education (years)	10.64	1.21	10.42	1.28	0.453	0.654
Illness duration (years)	9.50	5.63	–	–	–	–
Chlorpromazine Eq. (daily dosage/mg)	375.79	155.10	–	–	–	–
	***N***		***N***			
	
Gender (m/f)	10/4		10/4		–	–
Handedness (r/l)	12/2		12/2		–	–
Antipsychotic Med.	14		–		–	–
Co-medication^∗^	5		–		–	–
**BACS**						
Verbal memory	41.07	13.38	45.29	9.54	–0.959	0.346
Digit	19.21	4.30	20.00	4.67	–0.463	0.674
Motor	68.71	11.40	74.57	10.77	–1.397	0.147
Fluency	47.28	16.34	52.14	15.55	–0.806	0.428
Symbol coding	55.00	13.60	57.21	16.04	–0.394	0.697
ToL	17.43	2.50	17.29	2.33	–0.156	0.877
Total score	248.71	43.76	266.50	39.70	–1.126	0.270
**PANSS**						
Negative	19.07	3.56	–	–	–	–
Positive	17.14	2.77	–	–	–	–
General	38.29	3.69	–	–	–	–
Total score	74.50	7.43	–	–	–	–


*^∗^Co-medication of antipsychotics and mood stabilizers.*

### Experimental Design

**FIGURE 1:**
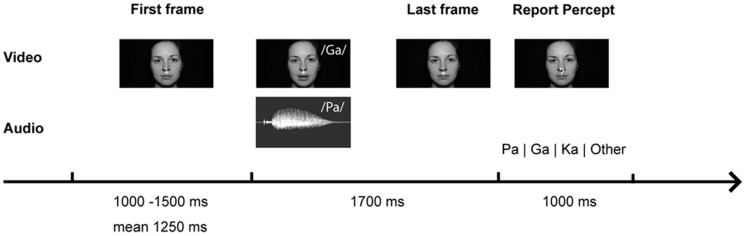
**McGurk trial with video frames of the syllable /ga/ and audio trace of the syllable /pa/.** Participants were presented with congruent (auditory /pa/ and visual /pa/) or incongruent (auditory /pa/ and visual /ga/) audiovisual syllables. They were instructed to reported which syllable they perceived.

The setup was identical to our study in healthy participants (Roa Romero et al., 2015). During the experiment different types of congruent and incongruent audiovisual syllable trials were presented (**Table 2** and **Supplementary Table S2**). Congruent syllable trials contained matching audiovisual syllables (e.g., visual /pa/ and auditory /pa/), whereas incongruent syllable trials contained non-matching audiovisual syllables (e.g., visual /pa/ and auditory /ka/). The congruent syllable combination visual /pa/ and auditory /pa/ served as control condition in the EEG data analysis. To induce the McGurk illusion, we presented the combination of a visual /ga/ and an auditory /pa/, which frequently led to the illusory perception /ka/ or “something else.” When the resulting perception of McGurk trials was /ka/ or “something else,” we will refer to these trials as ‘McGurk illusion trials.’ Importantly, the auditory syllable (i.e., /pa/) in congruent control trials and in McGurk trials was identical. In total 300 McGurk trials were presented. In addition, 150 incongruent syllable trials were presented (**Table 2**). These other incongruent syllables served as distractor stimuli to ensure that the McGurk illusion was specific to McGurk trials and not merely the result of the audiovisual mismatch. In each trial, the first frame of the video clip was presented for a random interval ranging from 1000 to 1500 ms (mean = 1250 ms). After the video clip, which had on average a duration of 990 ms (**Supplementary Table S2**), the last frame of the clip was presented on average for 710 ms. The total video sequence was presented for 1700 ms. Following the video clip, the last frame of each clip, in which the mouth of the actress was closed, was presented for 1000 ms. During this time the fixation cross turned into a question mark for 500 ms at a random time point and participants were required to indicate by a button press with the index, middle, ring or small finger of their right hand whether they had perceived the syllable /pa/, /ga/, /ka/, or “something else,” respectively. Each trial had a duration of 3700–4200 ms (**Figure 1**).

**Table 2:** Overview of presented congruent and McGurk-illusion trials.

Condition	Visual	Audio	*N*
Congruent^∗^	Pa	Pa	100
Congruent	Ga	Ga	100
Congruent	Ka	Ka	100
McGurk	Ga	Pa	300
Incongruent	Pa	Ga	30
Incongruent	Pa	Ka	30
Incongruent	Ga	Ka	30
Incongruent	Ka	Pa	30
Incongruent	Ka	Ga	30


*The middle panels show the visual and the auditory syllables. The remaining five incongruent syllables combinations comprised 150 trials. In total 750 trials were presented. ^∗^Congruent Pa–Pa trials served as control condition.*

### EEG Recording and Data Analysis

Electroencephalography data were recorded using a 128 channel active EEG system (EasyCap, Herrsching, Germany), which included two EOG electrodes (online: 1000 Hz sampling rate with a 0.016–250 Hz bandpass filter; offline: downsampling to 500 Hz, 1–125 Hz FIR bandpass filtering and 49.1–50.2 Hz, fourth order Butterworth notch filtering). To correct for EOG and ECG artifacts, independent component (IC) analyses were conducted (extended runica; Lee et al., 1999). On average 14.64 ± 0.82 (standard error of mean) ICs for ScZ patients and 16.71 ± 0.93 ICs for matched controls were rejected. Remaining noisy channels were interpolated using spherical interpolation (ScZ patients = 13.43 ± 0.88 channels; matched controls = 15.64 ± 1.03 channels). Epoched data were re-referenced to common average. For ERP analysis, data were filtered (2 Hz, second order and 35 Hz, 12th order two-pass Butterworth filter) and baseline corrected (–500 ms to –100 ms prior to sound onset). For the time-frequency analysis of lower frequency responses (i.e., 4–40 Hz) wavelet transformation with frequency depending Hanning window was computed in 2 Hz steps (time window Δt = 5/f, spectral smoothing: f = 1/Δt). For the analysis of higher frequency responses (i.e., 40–100 Hz) Slepian tapers (fixed time window *t* = 0.2 s, fixed spectral smoothing: *f* = 10 Hz) were applied. However, since we did not find robust modulations of high frequency (i.e., >40 Hz) responses in the current data, we focused the analysis to the frequency range from 4 to 40 Hz. Averaged oscillatory activity was baseline corrected (relative change, from –500 to –100 ms prior to sound onset).

The analysis of behavioral data focused on McGurk trials and congruent trials (**Table 3**). Reaction tendencies in McGurk trials were calculated as the relative proportion of illusion, audio, and visual percept responses (Keil et al., 2012). Independent *t*-tests between groups were conducted separately for rate of illusion, audio and visual percept (Bonferroni corrected α-level = 0.017).

**Table 3:** Means, standard deviation, and mean difference for behavioral performance (perceptual ratings in McGurk trials) in patients and controls.

	Patients		Controls		Statistics	
			
	Mean	*SD*	Mean	*SD*	*t*-values	*p*-values
Illusion percept %	78.30	20.36	65.07	29.09	1.395	0.175
Audio percept %	21.16	19.93	33.02	29.10	-1.259	0.219
Visual percept %	0.54	0.74	1.91	1.48	-1.516	0.141


*Each group comprised of 14 participants.*

The analysis of EEG data focused on the comparison of ERPs and oscillatory responses to McGurk illusion and congruent trials. The number of trials was equalized according to the lowest number of trials in either condition. On average, for each condition 62 ± 3.71 trials for ScZ patients and 69 ± 3.47 trials for matched controls were used. In order to examine whether any possible effects are driven by the incongruent audio–visual stimulation in McGurk trials (i.e., visual /ga/ and auditory /pa/) and not due to the multisensory fusion process that leads to the McGurk illusion, the same analyses for ERPs and oscillatory power were calculated for 19 ScZ patients and 19 matched controls, irrespective of McGurk illusion perception (see Supplementary Material and Supplementary Table S1). Hence, all McGurk trials, irrespective of perception were included in this analysis. Note that in this analysis two participants were excluded from each group due to insufficient quality of EEG data. Similar to our previous study (Roa Romero et al., 2015), we examined ERP amplitudes and oscillatory power at a medio-central region of interest (ROI), comprising 16 channels. The activity of the channels was averaged and served as dependent variable in the statistical analyses. In addition, we calculated the Global field power (GFP) for each Condition and Group as a measure of location-independent cortical activity integrating all channels (Esser et al., 2006). Due to a more complex factorial design we applied a different statistical analysis approach compared to our previous study, in which we computed non-parametric cluster statistics (Roa Romero et al., 2015). Specifically, for ERPs, GFP, as well as oscillatory power running 2 × 2 ANOVAs with the factors Group (ScZ patients vs. matched controls) and Condition (congruent vs. illusion) were conducted for each sample point (Schurger et al., 2008; Kissler and Koessler, 2011; Kissler and Herbert, 2013). In accordance with our previous study (Roa Romero et al., 2015), ERPs were analyzed in a time window from 0 to 500 ms and oscillatory responses from 0 to 850 ms following auditory syllable onset. The above-described 2 × 2-factorial ANOVA was conducted for each sample point in these intervals. To account for multiple testing, a time stability criterion of at least 10 consecutive significant sample points (i.e., 20 ms) was applied (Guthrie and Buchwald, 1991; Picton et al., 2000). For oscillatory responses the running 2 × 2-factorial ANOVA was computed for each sample point and frequency (4–40 Hz) in the 0 to 850 ms interval. Due to the low temporal resolution of the time-frequency transformation, a time stability criterion of at least 100 ms was applied. Significant main effects or interactions were followed-up by *t*-tests. Finally, Pearson correlations were computed between psychopathology scores (PANSS), McGurk illusion rate, and EEG data. To statistically control for the influence of antipsychotic medication, medication dosage was converted to chlorpromazine equivalent level (Gardner et al., 2010) and entered as covariate to partial correlation analyses in the patient group.

## Results

### Behavior

The recognition rate of congruent trials was at ceiling level (ScZ patients = 98.49%; matched controls = 97.92%). In McGurk trials ScZ patients and matched controls reported an illusory percept in 78.30 and 65.07% of trials, respectively, which was not significantly different [*t*(26) = 1.395, *p* = 0.175]. Moreover, the comparisons of the different percepts that could be evoked by McGurk trials did not reveal significant group differences (**Table 3**). We also examined whether there were behavioral differences between the samples of 19 participants per group for whom McGurk trials, irrespective of perception were analyzed. This comparison did also not reveal significant differences between ScZ patients (66.33%) and matched controls [53.14%; *t*(36) = 1.191, *p* = 0.242].

### Event-Related Potentials and Global Field Power

**FIGURE 2:**
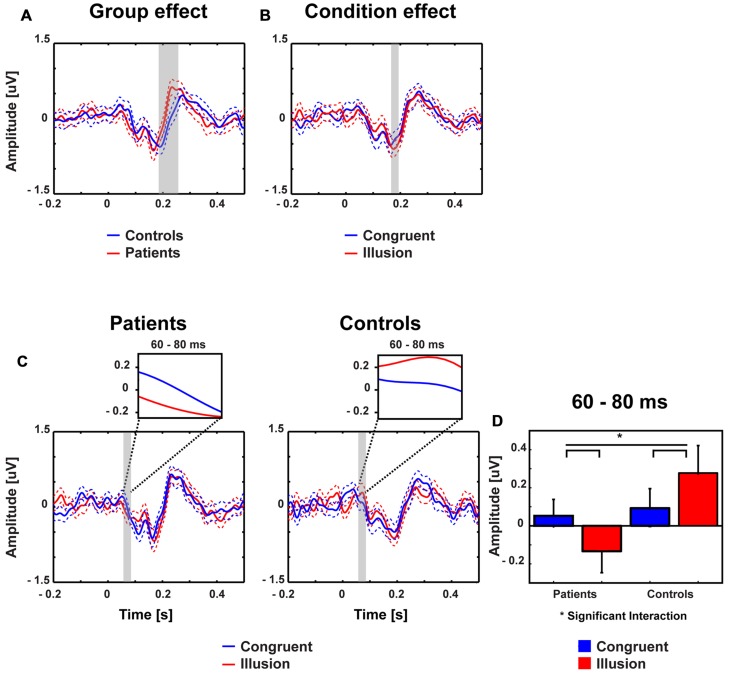
**Traces and amplitudes of early medio-central event-related potentials.** Traces of ERPs in patients (left) and controls (right) for illusion (red line) and congruent (blue line) trials. Time zero denotes the onset of the auditory syllable. Dashed lines represent standard error of mean. The significant time intervals are highlighted in gray. **(A)** Group effects were found between 190 and 250 ms. **(B)** Condition effects were found between 175 and 195 ms. **(C)** Interactions between group and condition were found after 60–80 ms. **(D)** Mean ERP amplitudes of the 60–80 ms time interval with error bars (standard error of mean). In patients amplitudes were more positive in congruent compared with illusory trials. By contrast, in the control group larger positive amplitudes were observed in illusory compared with control trials.

**FIGURE 3:**
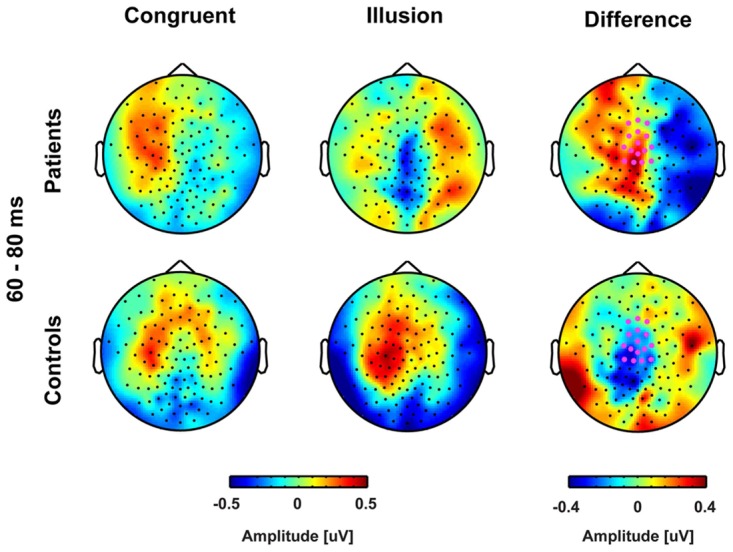
**Topographies of early event-related potentials for congruent and McGurk illusion trials.** The bold magenta dots in the right panel denote the electrode ROI for which significant group by condition effects in the time interval 60–80 ms were found.

Stimulus-evoked activity between McGurk illusion trials (i.e., McGurk trials in which participants reported an illusion) and congruent control trials was compared between 14 ScZ patients and 14 matched controls. The running 2 × 2 ANOVA revealed a significant main effect of Group between 190 and 250 ms [*F*(2,26) = 16.89, *p* = 0.0035], due to larger amplitudes in ScZ patients (0.275 μV) compared with matched controls (–0.160 μV). Furthermore, a main effect of Condition was found between 175 and 195 ms [*F*(2,26) = 8.06, *p* = 0.009], indicating larger negative amplitudes in illusion trials (–0.550 μV) compared with congruent trials (–0.370 μV). Notably, a significant Group by Condition interaction was observed between 60 and 80 ms [*F*(2,26) = 6.79, *p* = 0.015], indicating that the amplitude differences between illusion and congruent trials were significantly larger for matched controls than for ScZ patients (**Figures 2** and **3**). Follow-up *t*-tests for the 60 to 80 ms interval, which were conducted separately for each condition, revealed significant amplitude differences between groups in McGurk illusion trials [*t*(27) = 2.27, *p* = 0.04] but not in congruent trials [*t*(27) = 0.33, *p* = 0.74]. Furthermore, to examine the general effect of incongruence we compared ERPs for all McGurk trials, irrespective of subjective percept with the ERPs to congruent control trials. The running 2 × 2 ANOVA, which included 19 participants in each group, revealed no significant interactions or main effects of Group. However, main effects of Condition were found between 246 and 266 ms [*F*(2,36) = 8.12, *p* = 0.007] and between 338 and 368 ms [*F*(2,36) = 5.57, *p* = 0.023]. The first condition effect indicates larger positive amplitudes in congruent trials compared with McGurk trials. The latter condition effect showed the reversed pattern, indicated larger positive amplitudes in McGurk trials compared with congruent trials (**Supplementary Figure S1**).

The analysis of GFP for 14 ScZ patient and 14 matched controls revealed a significant main effect of Condition between 140 and 160 ms [*F*(2,26) = 10.16, *p* = 0.0037, **Supplementary Figure S4**], due to larger amplitudes in congruent compared with illusion trials. No other main effects or interactions were observed. In a final analysis step, we explored whether there are topographic differences in evoked activity between conditions and groups. To this end, within each subject, we calculated the Global Dissimilarity Index (GDI, Murray et al., 2008) between the congruent and illusion trials as a measure of difference in the topographies between both conditions. Subsequently, we compared the individual GDI values between groups with an independent *t*-test. This analysis did not reveal any significant effects in GDI, indicating that the topographies did not substantially differ between groups and conditions.

### Power of Oscillatory Activity

**FIGURE 4:**
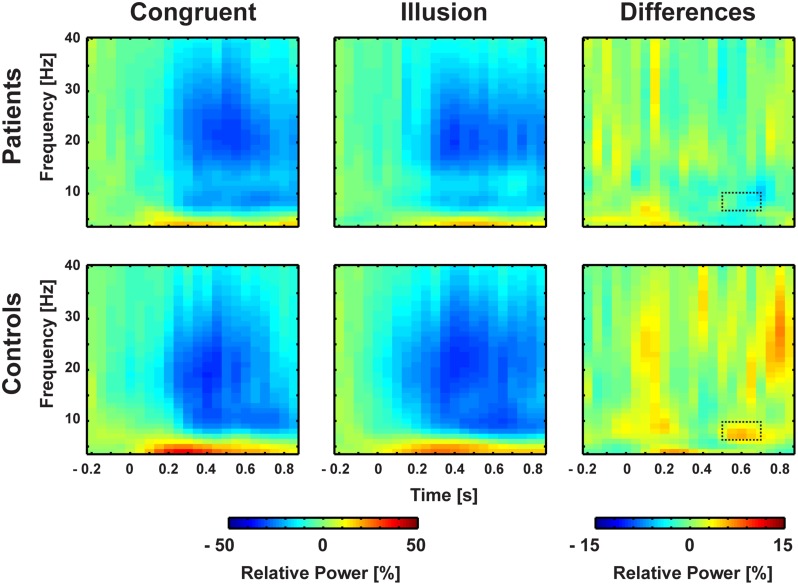
**Time-frequency responses of oscillatory responses at medio-central electrodes.** Time zero denotes the onset of the auditory syllable. Significant group by condition interactions were found in the alpha-band (8–10 Hz) after 550–700 ms. Significant cluster are marked by dashed line.

**FIGURE 5:**
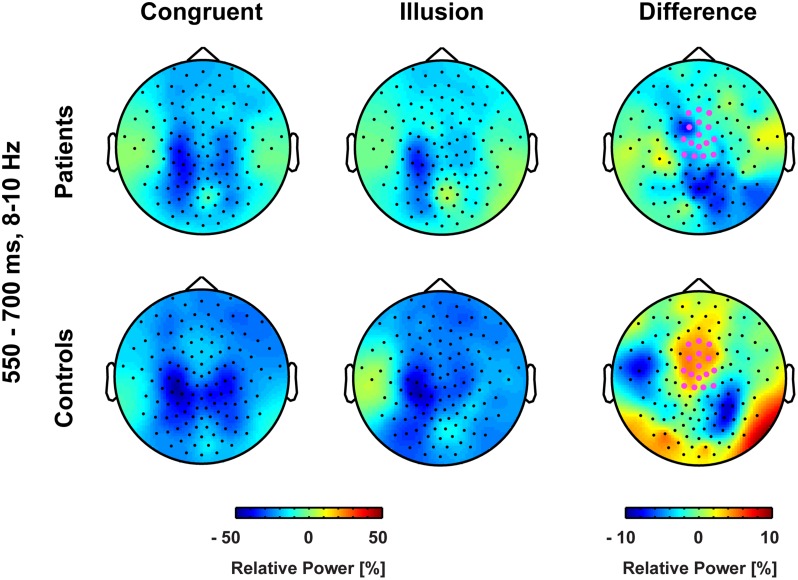
**Topographies of medio-central alpha-band power.** The bold magenta dots in the right panel denote the electrode ROI for which significant group by condition effects in the time interval 550–700 ms were found.

**FIGURE 6:**
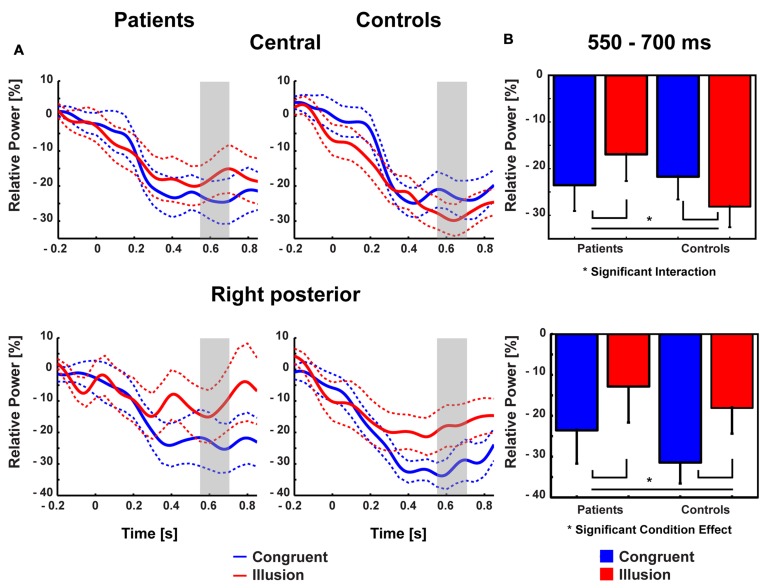
**Traces and amplitudes of medio-central alpha-band power.** **(A)** Traces of alpha-band power in patients (left) and controls (right) for illusion (red line) and congruent (blue line) trials at central (upper row) and right posterior (lower row) electrodes. Dashed lines represent standard error of mean. Interactions between group and condition were found after 550–700 ms indicated by gray trace. At right posterior electrodes a similar time course of alpha-band power was found. Time zero denotes the onset of the auditory syllable. Note that negative amplitude values represent stronger suppression. **(B)** Mean alpha-band power at medio-central (upper row) and right posterior (lower row) scalp regions in the 550–700 ms time interval with error bars (standard error of mean). In patients stronger alpha-band suppression was found in congruent compared with illusory trials. In controls stronger alpha-band suppression was observed in illusory compared with congruent trials. For right posterior alpha-band power both groups showed stronger suppression during congruent compared with illusion trials.

Aside from strictly time-locked event-related processes, the time-varying signatures of audiovisual processing of congruent and illusory percepts were of interest. The focus of this analysis was on oscillatory activity that differentiates between the varying percepts, although in both trial types (i.e., control and McGurk trials) identical auditory stimuli were presented. Therefore, oscillatory activity in response to control trials and McGurk illusion trials was compared. The running 2 × 2-factorial ANOVA, which was computed for each sample point in the 0–850 ms interval for the frequency range of 4–40 Hz, did not reveal significant main effects. However, a significant Group by Condition interaction was found in the alpha-band (i.e., 8–10 Hz) between 550 and 700 ms [*F*(2,26) = 5.47, *p* = 0.027; **Figure 4**]. In ScZ patients, alpha-band power was stronger suppressed in congruent (–0.24 μV^2^) compared with illusion trials [–0.17 μV^2^; *t*(13) = –1.80, *p* = 0.095; **Figure 5**]. The reversed pattern was observed in matched controls [illusion trials = –0.28 μV^2^, congruent trials = –0.22 μV^2^; *t*(13) = 1.64, *p* = 0.124]. Visual inspection of alpha-band power time course indicated that ScZ patients and matched controls primarily differed in the illusion condition. Following visual inspection of the alpha-band topography, we additionally explored possible alpha-band effects at right posterior electrodes (*n* = 7). For these electrodes we found a main effect of condition [*F*(2,26) = 7.20, *p* = 0.01] between 550 and 700 ms, which revealed less alpha-band power in congruent compared with illusion trials (**Figure 6**). Additionally, we investigated oscillatory power for McGurk trials, irrespective of subjective percept and congruent control trials. This analysis, in which 19 ScZ patients and 19 matched controls were entered, did not reveal significant interactions or main effects of Group. However, the ANOVA revealed a main effect of Condition in the theta-band (4 Hz) between 50 and 350 ms [*F*(2,36) = 8.04, *p* = 0.001]. In both groups, theta-band power was larger in congruent compared with McGurk trials, suggesting that incongruent visual information modulates early audiovisual processing (Supplementary Material and **Supplementary Figures S2** and **S3**).

### Relationships Between EEG Data, Illusion Rates, and Clinical Symptoms

**FIGURE 7:**
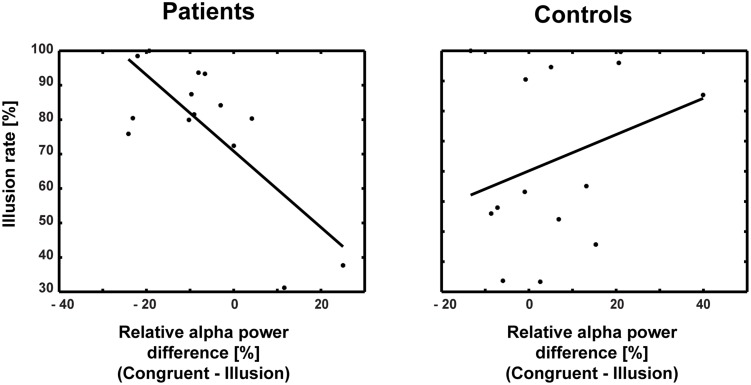
**Correlations between alpha-band power and illusion rate for patients and controls.** The correlation coefficients differed significantly between patients (*r* = –0.756) and controls (*r* = 0.30).

The correlations between ERP amplitudes and McGurk illusion rates for ScZ patients and matched controls were not significant. Moreover, the Bonferroni-corrected correlations between ERP amplitudes and alpha-band power were not significant (ScZ patients: *r* = –0.131, *p* = 0.671; matched controls: *r* = 0.441, *p* = 0.202). Additionally, none of the correlations between ERP amplitudes, alpha power and PANSS subscale scores were significant (all *p*-values >0.05). However, in ScZ patients a significant negative correlation between alpha-band power and illusion rate was found (partial correlation *r* = –0.756, *p* = 0.004). Interestingly, in matched controls there was a positive, yet not significant relationship between alpha-band power and illusion rate (*r* = 0.30, *p* = 0.29; **Figure 7**). The Pearson correlation coefficients differed significantly between groups (Z = 3.04, Zkrit = 1.65). Exploratory analysis of right posterior alpha-band power and illusion rate revealed no significant correlations for ScZ patients (*r* = –0.06, *p* = 0.85) and matched controls (*r* = –0.1, *p* = 0.74). Furthermore, the correlations between PANSS subscale scores and McGurk illusion rates were not significant.

## Discussion

In this electroencephalography study, we examined the McGurk illusion in ScZ. We observed altered ERPs and alpha-band suppression effects in ScZ patients compared with matched controls in McGurk illusion compared with congruent audiovisual syllable trials. Our behavioral analysis did not reveal group differences in McGurk illusion rates. Some studies have reported reduced illusion rates in ScZ (Gelder et al., 2002; White et al., 2014). However, in line with the present observation, other studies did not find group differences (Myslobodsky et al., 1992; Martin et al., 2013). The inconsistencies in findings could be due to differences in criteria for the definition of illusion rates, group ages, and heterogeneity of the clinical samples.

Our analysis of ERPs analysis revealed an early (60–80 ms) interaction at central electrodes. In matched controls, a larger positive deflection was found in illusion compared with congruent trials. By contrast, in ScZ patients, a larger negative deflection was found in illusion compared with congruent trials. The group differences in ERP amplitudes were primarily found in McGurk illusion trials, indicating altered early audiovisual processing of these trials in ScZ (**Figure 2**). A previous study in healthy participants has shown early processing differences between congruent and incongruent audiovisual syllables (Lebib et al., 2003). The authors found larger positive deflections for incongruent compared with congruent syllables. They suggested that the amplitude enhancement in incongruent syllables reflects an early detection of non-matching audiovisual information. Hence, the absence of early amplitude enhancement in McGurk illusion trials in ScZ patients might be due to a deficit in the early detection of non-matching audiovisual syllables. A study by Magnée et al. (2009), using an audiovisual P50 repetition-suppression paradigm, also revealed altered early ERPs in ScZ. Thus, our finding indicates altered early processing of sensory information across modalities in ScZ. Notably, when we compared congruent trials with all McGurk trials, independent of the subjective percept, there were no differences in early ERPs, neither in ScZ patients, nor in matched controls. Hence, the observation of early interaction effects in McGurk illusion trials indicates that there is specific processing deficit of these trials in ScZ patients. This assumption requires further empirical testing.

Another interesting finding in both groups were larger negative deflections (175–195 ms) in McGurk illusion compared with congruent trials. Moreover, the GFP also revealed differences between conditions at this latency. Previous studies in healthy participants found more negative auditory evoked P2 amplitudes for incongruent compared with congruent audiovisual syllables (Stekelenburg and Vroomen, 2007; Knowland et al., 2014). Similarly, another study using a McGurk oddball paradigm found a McGurk stimulus induced mismatch negativity at a similar latency (Saint-Amour et al., 2007). Hence, auditory evoked components could be a marker for congruency-detection and competition between sensory inputs during the processing of incongruent stimuli. A further finding was a larger positive deflection (190–250 ms) in ScZ patients compared with matched controls. Similar effects have been reported by Stekelenburg et al. (2013). The authors suggested that these larger ERPs could reflect multisensory processing deficits in the patient group. In summary, early ERPs in McGurk illusion trials in ScZ patients might be caused by a deficit in early incongruence detection in audiovisual syllables. In contrast, the results from late ERPs suggest no deficits in incongruence-detection but impaired mismatch-resolution during later processing stages of the McGurk illusion in ScZ.

Contrary to ERPs, the analysis of oscillatory responses did not reveal any early effects. The key finding in oscillatory responses was an interaction in long-latency (550–700 ms) alpha-band power: In ScZ patients medio-central alpha-band suppression was stronger in congruent compared with McGurk illusion trials. The pattern of suppression effects was reversed in the control group. Notably, the time course of medio-central alpha-band suppression in congruent trials was similar in both groups. By contrast, in illusion trials the suppression of later medio-central alpha-band power was more pronounced in matched controls compared with ScZ patients (**Figure 6**). This could reflect a lower signal-to-noise ratio in the auditory system during the processing of McGurk illusion in ScZ patients. Klimesch et al. (2007) and Klimesch (2012), hypothesized that suppression of alpha-band power is a neural signature of active processing in task relevant networks. Hence, the stronger alpha-power suppression presumably indicates a better signal-to-noise ratio, because irrelevant information (noise) is inhibited. In matched controls we found stronger medio-central alpha-band suppression in McGurk illusion compared with congruent trials. In contrast, ScZ patients showed less medio-central alpha-band suppression in illusion trials, which could indicate impaired integrative processing. Further, the reduced medio-central alpha-band power suppression in ScZ patients during illusion trials indicates a state, in which irrelevant information is not appropriately inhibited and the signal to noise ratio in the auditory system during the processing of McGurk illusion might be lower. Alpha-band suppression effects have been recently found in auditory illusion paradigms (Müller et al., 2013; Leske et al., 2014). Müller et al. (2013) observed a positive relationship between alpha-band suppression and illusory perception of music in an auditory continuity paradigm. Hence, the altered alpha-band suppression in ScZ patients might reflect abnormal processing of the auditory aspect of the McGurk illusion.

The medio-central topography of the alpha-band effect indicates an involvement of auditory and/or frontal structures. Auditory oddball tasks (Koh et al., 2011) and auditory gating paradigms (Popov et al., 2011) revealed altered alpha-band suppression in ScZ. In addition, alpha-band power modulations in the visual cortex have been found to contribute to multisensory illusions, such as the sound-induced flash illusion (Lange et al., 2014). Low alpha-band power indicates the increased excitability of visual areas and determines stimulus perception by regulating the incoming flow of information, within and between sensory areas, such as visual and auditory cortex.

Interestingly, in our study alpha-band power suppression over right posterior areas was stronger in congruent compared with McGurk illusion trials. This effect in right posterior alpha-band power was similar in both groups, indicating intact processing of McGurk illusion trials in visual areas in ScZ patients (**Figure 6**). Thus, the processing of McGurk illusion trials in ScZ seems to be specifically altered in auditory and/or frontal areas. The less pronounced medio-central alpha-band suppression in illusion trials could mirror reduced auditory processing, possibly due to an increased ambiguity in the encoding of auditory information. In contrast, in matched controls there might be a stronger processing of both auditory and visual stimuli in the illusion trials. As shown in this study, the modulations in alpha-band power presumably mirror a process that differentiates between patients and matched controls during the formation of the McGurk illusion.

In a previous study, we investigated the McGurk illusion in healthy subjects and revealed modulations in late beta-band activity over left temporal and frontal areas (Roa Romero et al., 2015). We suggested that the suppression of late beta-band power fosters the formation of a coherent, subjectively congruent percept, namely the McGurk illusion. The absence of differences in the late beta-band power in the present study could indicate that the process of perception formation itself is not altered in ScZ patients. This could lead to identical behavior reflected by similar illusion rates between both groups.

Another finding in our study was that the medio-central alpha-band suppression in ScZ patients was negatively correlated with the McGurk illusion rate, while it tended to be positively associated with the illusion rate in matched controls. This supports the notion that less pronounced alpha-band suppression in McGurk illusion in ScZ patients reflects altered multisensory integration. Moreover, we found no correlation between the effects of early ERP and late alpha-band power. This indicates that the effects might reflect distinct aspects of audiovisual processing in the McGurk illusion.

In this study, we obtained oscillatory responses in the EEG, but did not find clear modulations in gamma-band oscillations. MEG compared to the EEG has a higher sensitivity in the measurement of high frequency oscillations (Muthukumaraswamy and Singh, 2013). Additionally, numerous studies have revealed reduced gamma-band power in ScZ patients (Gallinat et al., 2004; Leicht et al., 2010). These factors might have contributed to the absence of gamma-band modulations in the present study. It would be interesting to use MEG to examine gamma-band oscillations during the processing of the McGurk illusion in ScZ patients, to uncover the possible role of gamma-band power for illusory perception.

## Conclusion

Taken together, our study revealed altered early and late processing of McGurk illusion trials in ScZ. The early ERP effect might reflect audiovisual processing deficits in ScZ patients. The altered late alpha-band suppression effects could reflect abnormal multisensory integration in auditory and/or frontal areas. Our study provides new insight into the processing of the McGurk illusion in ScZ and fosters the notion that alpha-band oscillations reflect altered multisensory integration in ScZ patients.

## Author Contributions

YRR, JK, and DS designed the experiment. YRR and JB recruited the patients and healthy controls and collected the data. YRR performed data analysis and prepared MS. JK assisted data analysis and manuscript preparation. DS, JG, and MN reviewed and edited the MS.

## Conflict of Interest Statement

The authors declare that the research was conducted in the absence of any commercial or financial relationships that could be construed as a potential conflict of interest.
