# Supplementary material for: Alpha-Band Oscillations Reflect Altered Multisensory Processing of the McGurk Illusion in Schizophrenia
Source: Front Hum Neurosci. 2016 Feb 12;10:41. doi: 10.3389/fnhum.2016.00041 (PMC4751891; doi:10.3389/fnhum.2016.00041)
Supplement: Supplementary file 1 [file Presentation_1.PDF]

Supplementary Material

Roa-Romero et al. Alpha-band Oscillations reflect altered multisensory processing of the McGurk Illusion in Schizophrenia.

Supplementary Methods

Participants included in the analysis of all McGurk trials

Since all McGurk trials, irrespective of perception, were used for the analyses only two participants per group were excluded due to an insufficient number of EEG trials after artifact rejection. We computed the same analyses of ERPs and oscillatory power as described in the main article. The analyses included 19 patients (7 female, mean age = 34.58 y) and 19 controls (5 female, mean age = 34.37 y). Supplementary Table 1 provides an overview on the demographic data, cognitive performance, and clinical scores.

Supplementary Results

Comparison of McGurk illusion rates between 19 patients and 19 controls that were included in the EEG data analysis of all McGurk trials

The illusion rates between ScZ patients (66.33 %) and matched controls (53.14 %;) did not significantly differ ($t(36) = 1.191$, $p = 0.242$).

Analysis of EEG data to all McGurk trials

The running 2 x 2 ANOVA for the ERPs in response to all McGurk trials (i.e. independent of the percept) revealed a significant main effect of Condition between 246 and 266 ms ($F(2,36) = 8.12$, $p = 0.007$) and between 338 and 368 ms ($F(2,36) = 5.57$, $p = 0.023$). The 246 to 266 ms effect of Condition indicates larger positive amplitudes in congruent trials compared with McGurk trials. The latter effect of Condition showed the reversed pattern: larger positive amplitudes in McGurk trials compared with congruent trials (Supplementary Figure 1). No significant interactions or main effects of Group were found. The running 2 x 2 ANOVA for oscillatory responses revealed a main effect of Condition in the theta-band (4 Hz) between 50 and 350 ms ($F(2,36) = 8.04$, $p = 0.001$). In both groups, theta-band power was larger in congruent compared with McGurk trials, suggesting that incongruent visual information modulates early audiovisual processing (Supplementary Material and Supplementary Figures S2 and S3). No significant interactions or main effects of Group were observed.

Supplementary Discussion

Outcome of the analysis of all McGurk trials

For congruent control and McGurk trials we found no significant interactions and no main effects of Group in ERPs, which survived the correction for multiple comparison (Figure S1). However, we found two significant main effects of Condition between 246 and 266 ms and 338 and 368 ms. The main effect in the 246 to 266 ms interval indicated larger positive amplitudes in congruent trials compared with McGurk trials. The latter effect in the 338 to 368 ms interval had the reversed pattern, indicated larger positive amplitudes in McGurk trials compared with congruent trials. Similarly to our late ERP effect, Arnal et al. (2011) found increased amplitudes of event-related fields during the presentation of incongruent compared to congruent audiovisual syllables. This suggests a longer latency processing of audiovisual stimulus incongruence.

Analogous to the analysis of oscillatory responses in McGurk illusion trials, we found no significant interactions and no main effects of Group. However, we found a significant main effect of Condition in the theta-band (4 Hz) power between 50 and 350 ms (see Figure S2 & S3). This effect was due to an enhanced theta-band power for congruent compared with McGurk trials, which was similarly observed in both groups. Interestingly, the theta-band effect occurred in a similar latency and in a similar frequency as a previously reported by Lange et al. (2013). Lange and colleagues investigated the processing of congruent and incongruent audiovisual speech vowels. In this study, theta-band power between 50 and 250 ms was increased during the presentation of incongruent compared to congruent audiovisual speech. Albeit the direction of the theta-band effects differed between the present and the Lange et al. (2013) study, the effects in early theta-band power suggests that incongruent visual information modulates early audiovisual processing.

References

Arnal, L.H., Wyart, V., & Giraud, A.-L. (2011) Transitions in neural oscillations reflect prediction errors generated in audiovisual speech. *Nat. Neurosci.*, **14**, 797–801.

Lange, J., Christian, N., & Schnitzler, A. (2013) NeuroImage Audio – visual congruency alters power and coherence of oscillatory activity within and between cortical areas. *Neuroimage*, **79**, 111–120.

Supplementary Figure S1

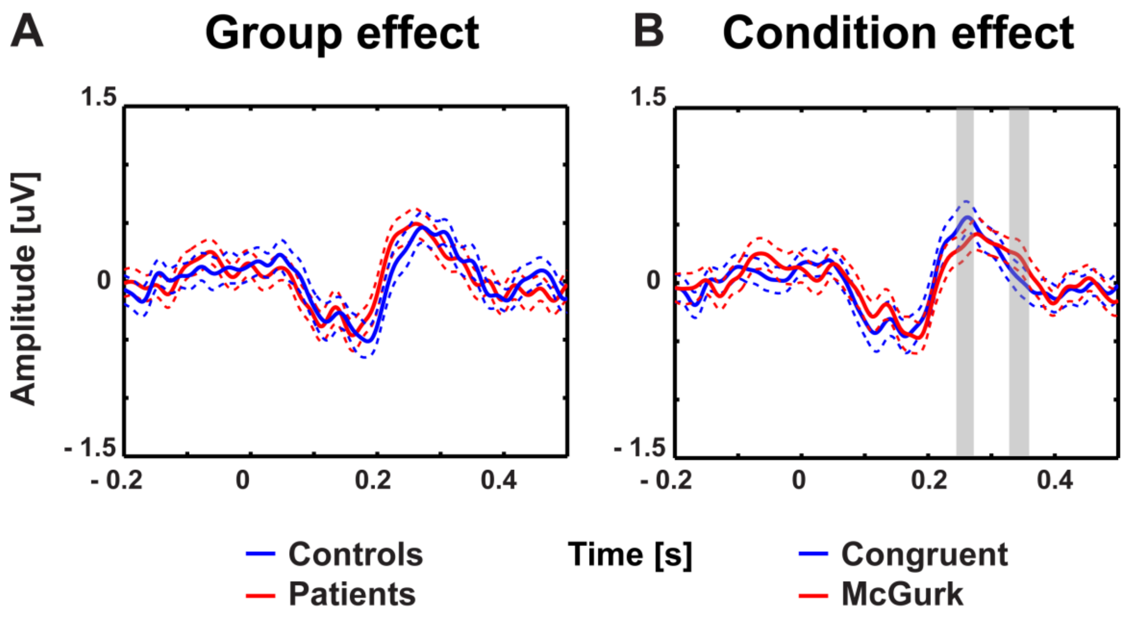

Figure S1. A) Traces of medio-central event-related potentials for all ScZ patient (red line), and matched controls (blue line). Dashed lines reflect traces with standard errors of mean. There were no differences between groups and no interaction. B) Traces of medio-central event-related potentials for all McGurk trials (red line), irrespective of perception, and congruent control trials (blue line). Dashed lines reflect traces with standard errors of mean. The analysis revealed main effects of condition at 246 to 266 ms and 338 to 368 ms marked with gray traces.

Supplementary Figure S2

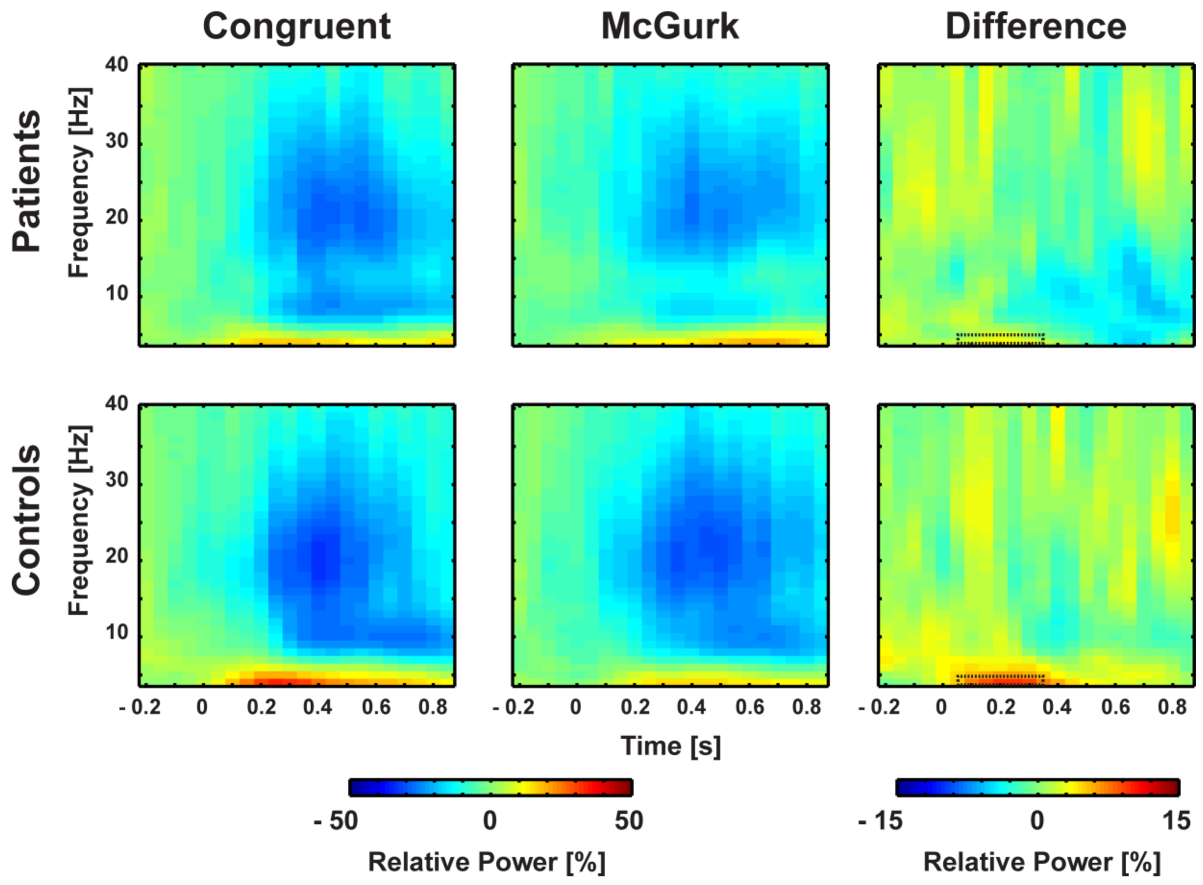

Figure S2. Time-frequency planes of oscillatory responses at medio-central electrodes. The dashed squares highlight the significant theta-band condition effect at 50 to 350 ms. Time zero denotes the onset of the auditory syllable.

Supplementary Figure S3

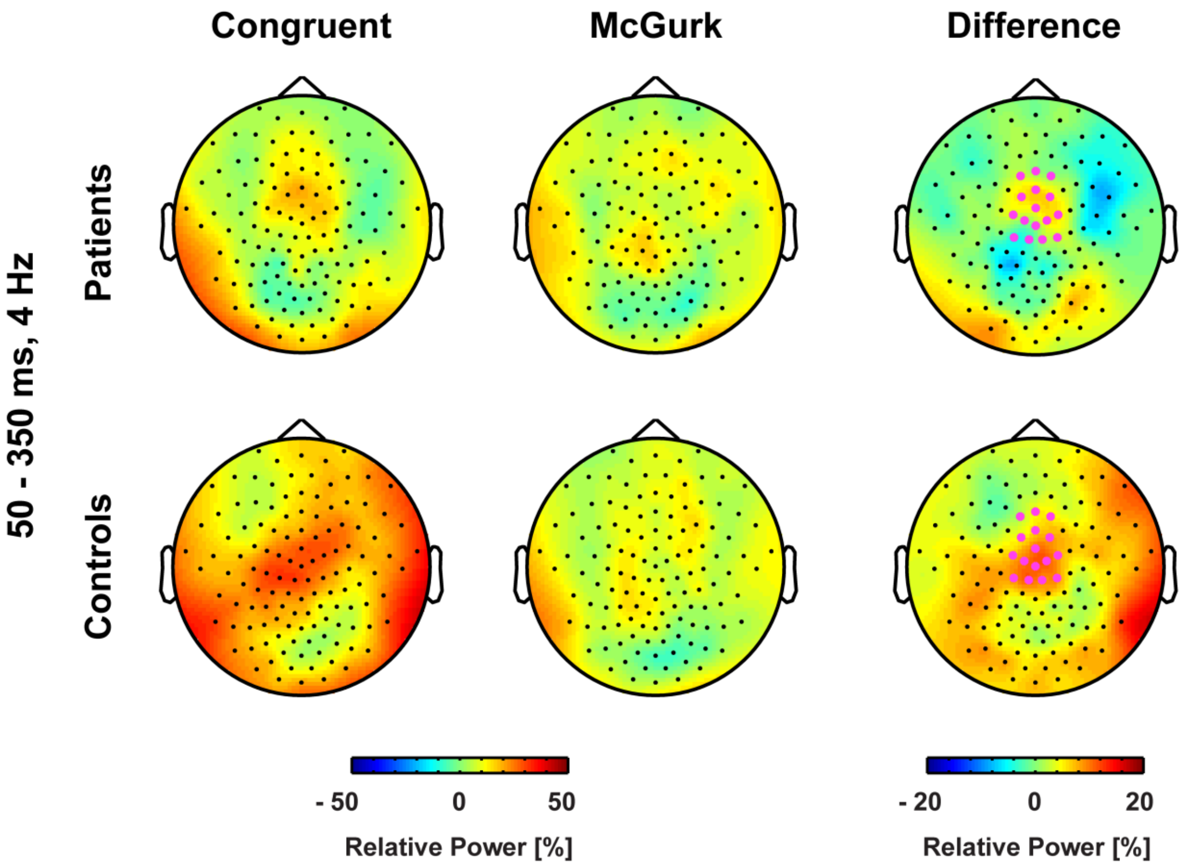

Figure S3. Topographies of medio-central theta-band power in the 550 to 700 ms interval. The bold magenta dots in the right panel denote the medio-central electrode group.

Supplementary Figure S4

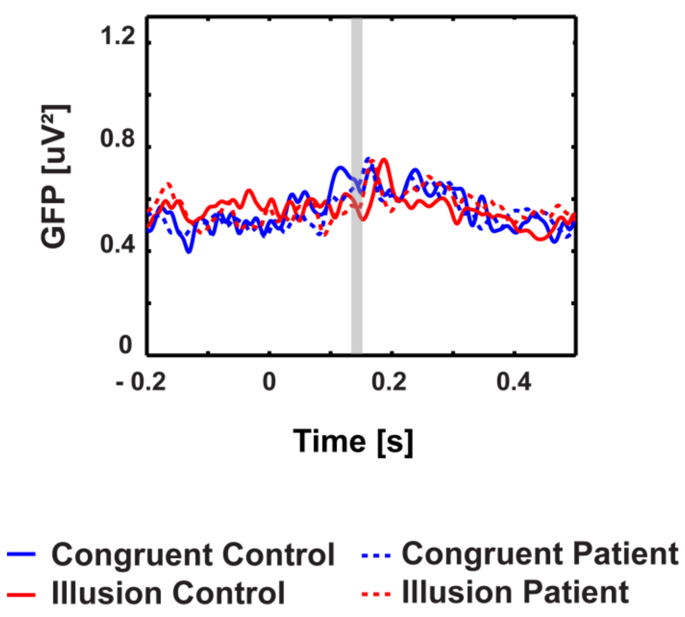

Figure S4. Global field power traces for patients (dashed lines) and matched controls (solid lines) for McGurk illusion trials (red lines), and congruent control trials (blue lines). Significant condition effect was found after 140-160 ms. Significant time interval are marked by gray shading. There were no further differences between groups and no interaction between these factors.

Supplementary Table 1

	Patients		Controls		Statistics	
	**Mean**	**SD**	**Mean**	**SD**	**t-values**	**p-values**
Age (years)	33.63	6.77	34.37	7.46	-.319	.752
Education (years)	10.95	1.58	10.95	1.72	0	1.00
Illness duration (years)	9.84	4.84	-	-	-	-
Chlorpromazin Eq. (daily dosage/ mg)	381.71	184.62	-	-	-	-
	**N**		**N**			
Gender (m/f)	12/7		14/5		-	-
Handedness (r/l)	15/4		16/3		-	-
Antipsychotic Med.	19		-		-	-
Co-medication*	5		-		-	-
**BACS**						
Verbal Memory	43.74	13.24	46.89	9.95	-.831	.411
Digit	19.74	4.28	20.11	4.41	-.261	.795
Motor	67.79	11.58	75.37	10.03	-2.52	.038
Fluency	48.26	14.50	52.90	14.66	-.979	.334
Symbol coding	56.68	13.45	57.84	14.42	-.256	.799
ToL	17.89	2.60	17.58	2.27	.399	.692
Total score	254.10	42.31	270.68	37.95	-1.27	.212
**PANSS**						
Negative	18.63	3.30	-	-	-	-
Positive	16.68	2.79	-	-	-	-
General	38.11	3.33	-	-	-	-
Total score	73.42	6.96	-	-	-	-

Table 1. Overview of demographic data. *Co-medication of antipsychotics and mood stabilizers

Supplementary Table 2

Syllable	/Pa/	/Ka/	/Ga/
Place of articulation	bilabilal	velar	velar
Visual duration	858 ms	1221 ms	891 ms
Auditory duration	329 ms	429 ms	348 ms
Latency difference between visual motion onset and auditory onset	337 ms	594 ms	429 ms

Table 2. Overview of the stimulus durations of the presented syllables and differences between visual motion onset and auditory syllable onset. Note that the latency difference between visual motion onset and auditory onset refers to congruent audiovisual syllable combinations (e.g., visual /Pa/ and auditory /Pa/).
